# Predictive ability of the Chinese visceral adiposity index for incident hypertension in working-aged Koreans

**DOI:** 10.4178/epih.e2024034

**Published:** 2024-02-27

**Authors:** Ju Young Jung, Chang-Mo Oh, Hyun chul Jo, Sung Keun Park

**Affiliations:** 1 Total Healthcare Center, Kangbuk Samsung Hospital, Sungkyunkwan University School of Medicine, Seoul, Korea; 2 Departments of Preventive Medicine, Kyung Hee University School of Medicine, Seoul, Korea; 3 Departments of Internal Medicine, Jo Hyun Chul Private Clinic, Gimpo, Korea; 4 Center for Cohort Studies, Kangbuk Samsung Hospital, Sungkyunkwan University School of Medicine, Seoul, Korea

**Keywords:** Chinese visceral adipose index, Visceral adipose index, Body mass index, Waist circumference, Hypertension

## Abstract

**OBJECTIVES:**

The Chinese visceral adiposity index (CVAI) was developed to assess visceral adipose tissue in the Asian population. This study evaluated the predictive ability of the CVAI for incident hypertension in Korean adults.

**METHODS:**

The study participants included 128,577 Koreans without hypertension. They were grouped in quartiles according to body mass index (BMI), waist circumference (WC), visceral adipose index (VAI), and CVAI values. The Cox proportional hazard assumption was used to evaluate the hazard ratio (HR) and 95% confidence interval (CI) for incident hypertension (adjusted HR [95% CI]) according to quartile level across a follow-up period of 6.9 years. Subgroup analyses were conducted by gender and obesity. The area under the curve was calculated to compare the predictive abilities of all indices (BMI, WC, VAI, and CVAI) for incident hypertension.

**RESULTS:**

The CVAI was proportionally associated with the risk of hypertension in all participants (quartile 1: reference; quartile 2: 1.71 [95% CI, 1.59 to 1.82]; quartile 3: 2.41 [95% CI, 2.25 to 2.58]; and quartile 4: 3.46 [95% CI, 3.23 to 3.71]). Time dependent receiver operating characteristic curve analysis indicated that the CVAI was superior to BMI, WC, and VAI in predicting hypertension at the 2-year, 4-year, 6-year, and 8-year follow-ups. This finding was also observed in the gender and obesity subgroups. The predictive ability of the CVAI was greater in the women and non-obese subgroups than in the men and obese subgroups.

**CONCLUSIONS:**

The CVAI was a stronger predictor of hypertension than BMI, WC, and VAI.

## GRAPHICAL ABSTRACT

[Fig f1-epih-46-e2024034]

## Key Message

Hypertension is a leading risk factor for cardiovascular disease. It is known that visceral adiposity has an important role in the pathogenesis of hypertension in obesity. Recent studies have demonstrated that Chinese visceral adiposity index (CVAI) is more discriminative the high risk group for cardiovascular disease in Asians than other obesity indices. We compared the predictive ability for the development hypertension among body mass index, waist circumference, visceral adiposity index, and CVAI. Our study showed that CVAI is superior to predict hypertension than other indices.

## INTRODUCTION

Hypertension is a leading preventable risk factor for cardiovascular (CV) disease and all-cause mortality worldwide [1,2]. Despite improvements in the therapeutic approach to hypertension in recent decades, a significant number of hypertensive patients are still poorly controlled [3]. Thus, to prevent hypertension and reduce the disease burden, it is imperative to use accurate and applicable clinical indices to distinguish high-risk groups.

The relationship between obesity and hypertension is well established. A meta-analysis showed a 1-fold to 2-fold increased risk of incident hypertension with increases in obesity indices including body mass index (BMI), waist circumference (WC), waist‐to‐hip ratio, and waist‐to‐height ratio [4]. In particular, abdominal obesity (i.e., abdominal fat deposition) was found to be an important risk factor for cardiometabolic diseases including hypertension. It has been demonstrated that abdominal obesity is more often associated with CV risk than general obesity in adults [5,6].

Visceral adipose tissue (VAT) is a major factor mediating the association between abdominal obesity and increased CV risk. Previous studies have indicated that visceral fat is more closely linked to the development of hypertension than subcutaneous fat [7,8], raising interest in VAT as a reliable predictor of hypertension. The visceral adiposity index (VAI) is a mathematical model that allows inexpensive and precise assessment of visceral adiposity by clinical and anthropometric measurements [9]. Cross-sectional studies have demonstrated that the VAI is significantly associated with hypertension among Caucasians [10,11]. However, the VAI may have drawbacks when assessing body fat distribution in Asians because it was designed for Caucasians.

The Chinese visceral adiposity index (CVAI) was developed for Chinese adults using demographic, anthropometric, and metabolic characteristics [12]. Studies have suggested that the CVAI is superior to the VAI and other classic indices for obesity in predicting cardiometabolic disease including diabetes mellitus (DM) [13,14] and atherosclerotic diseases [15,16] among Chinese and Japanese populations. In addition, the CVAI has been significantly associated with the risk of hypertension in both cross-sectional [17] and prospective studies [18] of middle-aged Chinese. However, there is still insufficient data to confirm the association between the CVAI and the risk of hypertension in Asians. Furthermore, published studies have been conducted among Chinese populations only.

To validate the predictive ability of the CVAI for hypertension in Asians other than Chinese, we evaluated the risk of incident hypertension according to the CVAI quartile levels in 128,577 Koreans. In addition, we compared the CVAI, BMI, WC, and VAI as predictors of hypertension to identify any comparative advantage of the CVAI.

## MATERIALS AND METHODS

### Study participants and exclusion criteria

Relevant clinical and socio-demographic data were obtained from the Kangbuk Samsung Health Study (KSHS). The KSHS is a cohort study to investigate the medical data of Koreans who have received medical health check-ups in Kangbuk Samsung Hospital. Korea’s Industrial Safety and Health law requires that all Korean employees receive a medical health check-up annually or biennially. We initially enrolled 221,954 adults aged 18 years to 87 years who underwent medical check-ups between March 2011 and December 2012. From this sample, 65,211 participants were excluded for the following reasons: 852 participants were missing values for age, BMI, WC, triglyceride (TG), and high density lipoprotein-cholesterol (HDL-C) levels required to assess the VAI and CVAI; 36,167 participants were missing values for clinical parameters (e.g., fasting glucose, history of hypertension, homeostatic model assessment for insulin resistance [HOMA-IR]); 3,850 participants were taking medication for dyslipidemia; 5,778 participants had a history of major diseases including cancer, stroke, and coronary artery disease; and 18,564 participants had a history of baseline hypertension. Of the remaining 156,743 participants, we finally included the 128,577 participants who had revisited the hospital to receive health check-ups between January 2013 and December 2019.

### Clinical, anthropometric, and biochemical data

Past medical history and health-related behaviors were confirmed through a self-administered questionnaire, and anthropometric measurements such as weight, WC, and height, as well as biochemical tests including a lipid profile and fasting glucose were performed on all participants. Smoking patterns were divided into 3 categories: never, former, and current smoker. Alcohol consumption and frequency were assessed by the self-administered questionnaire. All participants were asked to answer questions about their average drinking frequency (day/wk or day/mo) and amount (grams). The average frequency and amount of alcohol intake was converted into daily alcohol use (g/day). The degree of physical activity was evaluated by the Korean-validated version of the International Physical Activity Questionnaire (IPAQ) short form and classified into 3 categories (low, moderate, and high) according to the guidelines prescribed by the IPAQ core group (http://www.ipaq.ki.se). Hypertension was defined as a prior diagnosis of hypertension or having a measured blood pressure (BP) ≥ 140/90 mmHg at initial and follow-up examinations. The cut-off point for hypertension was based on the Korean Hypertension Society guidelines [19]. Each BP was measured 3 times in a sitting position after a 5-minute rest by a trained nurse using an automated device with an interval of at least 30 seconds (53000- E2; Welch Allyn, Skaneateles Falls, NY, USA). Final BP levels were an average of the second and third BP measurements. The BMI was calculated by dividing weight (kg) by the square of the height (m^2^). Obesity was defined as a BMI ≥ 25 kg/m^2^ according to the International Obesity Task Force recommendation [20]. WC was measured at the narrowest point between the lower border of the rib cage and the iliac crest during minimal respiration in an erect position. DM was defined as one of following conditions: fasting glucose ≥ 126 mg/dL, glycated hemoglobin (HbA1c) ≥ 6.5%, or a prior diagnosis of DM [21].

Blood samples were collected after > 12 hours of fasting and were drawn from an antecubital vein. The fasting serum glucose was measured by the hexokinase method, and the HbA1c was measured by immunoturbidimetric assay with a Cobra Integra 800 automatic analyzer (Roche Diagnostics, Basel, Switzerland).

The enzymatic colorimetric test was used to measure total cholesterol and TG levels. Low-density lipoprotein cholesterol (LDL-C) and HDL-C were measured using the homogeneous enzymatic colorimetric test and the selective inhibition method, respectively (Advia 1650 Autoanalyzer; Bayer Diagnostics, Leverkusen, Germany). The VAI was calculated using the following equations [9]:


Men: WC/(39.68+[1.88× BMI])× (TG/1.03)× (1.31/HDL-C)Women: WC/(36.58+[1.89× BMI])× (TG/0.81)× (1.52/HDL-C)

The CVAI was calculated using the following equations [12]:


$$\text{Men:}-267.93+0.68\times \mathrm{age}+0.03\times \mathrm{BMI}+4.00\times \mathrm{WC}+22.00\times {\mathrm{Log}}_{10}\mathrm{TG}-16.32\times \mathrm{HDL}-C\;\text{Women:}-187.32+1.71\times \mathrm{age}+4.23\times \mathrm{BMI}+1.12\times \mathrm{WC}+39.76\times {\mathrm{Log}}_{10}\mathrm{TG}-11.66\times \mathrm{HDL}-C\;\text{(TG and HDL-C levels were calculated in mmol/L)}$$

Detailed descriptions of the study population and data collection are included in a previous study by our group [22].

### Statistical analysis

To analyze baseline clinical characteristics, all participants were divided into 4 categories based on CVAI quartile levels. The baseline parameters of the groups are presented as means± standard deviations for continuous variables and as proportions for categorical variables. We compared variable differences among the 4 groups, using analysis of variance for continuous variables and the chi-square test for categorical variables.

A Cox proportional hazard assumption was used to calculate the unadjusted and multivariable-adjusted hazard ratio (HR) and 95% confidence interval (CI) for hypertension. The multivariable adjusted HR (95% CI) was calculated for the quartile groups of BMI, WC, VAI, and CVAI. The adjusting covariates were the major risk factors of hypertension (model 1: age, gender, physical activity, alcohol intake, smoking, and DM), and the indicators of insulin resistance and chronic inflammation (model 2: model 1 plus HOMA-IR and c-reactive protein [CRP]). The incidence of cases, the incidence density (incident cases per 1,000 person-years [PYs]), and the PYs of each group were calculated. The proportional hazards assumption was confirmed by log-log plots and the Schoenfeld residual test. To verify multicollinearity between variables, we used variance inflation factor (VIF) analysis, which confirmed that there were no variables with a VIF >10. Subgroup analysis was performed according to gender and the presence or absence of obesity. Obesity subgroups were determined by a BMI ≥ 25 kg/m^2^ (obese subgroup) or a BMI < 25 kg/m^2^ (non-obese subgroup).

Time dependent receiver operator characteristic (ROC) analysis was performed to compare the abilities of BMI, WC, VAI, and CVAI to predict the development of hypertension across the follow-up period. Because the maximum follow-up period was 8.8 years, the area under the ROC curve (AUC) and 95% CI were calculated based on the incidence of hypertension at 2 years, 4 years, 6 years, and 8 years. BMI, WC, VAI, and CVAI were treated as continuous variables. A p-value was also calculated in each time dependent AUC (reference: BMI). The time dependent ROC analysis was calculated using the TimeROC package (Comprehensive R Archive Network [CRAN], R Foundation for Statistical Computing, Vienna, Austria). Since time dependent ROC analysis required a significant amount of computing power and memory, 10% of the subjects were randomly selected from the total and from each subgroup (men, women, obese, and non-obese) and included in the time dependent ROC analysis.

All statistical analyses were performed using R version 4.1.3 (R Foundation for Statistical Computing), and a value of p< 0.05 (2-sided) indicated statistical significance in all analyses.

### Ethics statement

Ethics approval for the study protocol and analysis of the data were obtained from the Institutional Review Board (IRB) of Kangbuk Samsung Hospital (IRB No. KBSMC 2022-08-041). The IRB of Kangbuk Samsung Hospital waived informed consent for the study because only retrospective data was assessed, and all personal information was anonymized.

## RESULTS

Baseline clinical characteristics of the study participants according to CVAI quartile are described in Table 1. Our study participants were working-aged adults with an average age of 38.4± 6.5 years, and a majority were men (58.6%). The proportion of men was only 14.8% in quartile 1, but reached 93.3% in quartile 4. The proportion of men, ages, BPs, TG levels, HOMA-IR levels, average alcohol consumption, and current smokers, as well as the prevalence of DM all increased with each subsequent quartile level of the CVAI. During the 6.9-year median follow-up period, 18,503 cases of hypertension (14.4%) were newly diagnosed among 128,577 study participants. The incidence of hypertension also increased with the quartile levels of the CVAI.

The multivariable adjusted HR (95% CI) for hypertension revealed the proportional relationship between the CVAI quartile levels and the risk of hypertension among all study participants (quartile 1: reference; quartile 2: 1.71 [95% CI, 1.59 to 1.82]; quartile 3: 2.41 [95% CI, 2.25 to 2.58]; and quartile 4: 3.46 [95% CI, 3.23 to 3.71]) (Table 2). An identical relationship pattern was observed in the analysis for BMI, WC, and VAI. While the CVAI showed the greatest HR for hypertension, the VAI showed the lowest HR (quartile 1: reference; quartile 2: 1.26 [95% CI, 1.20 to 1.33]; quartile 3: 1.52 [95% CI, 1.45 to 1.59]; and quartile 4: 1.93 [95% CI, 1.84 to 2.02]).

Subgroup analysis was conducted by gender and obesity (BMI ≥ 25 kg/m^2^). In the subgroup analysis for gender (Table 3), the risk of hypertension was proportionally associated with the quartiles of BMI, WC, VAI, and CVAI in both men and women. The magnitude of the HR for hypertension was greatest in the CVAI, and lowest in the VAI for each quartile group. The HR for hypertension was higher for women than men in each quartile group of the CVAI. Obesity subgroup analysis showed that the quartile levels of WC, VAI, and CVAI were proportionally associated with the risk of hypertension in both the obese subgroup (BMI ≥ 25 kg/m^2^) and the non-obese subgroup (BMI < 25 kg/m^2^) (Table 4). The CVAI had a higher HR for hypertension in each quartile group than WC and VAI. The non-obese subgroup showed a higher HR for hypertension in each quartile group of the CVAI than the obese subgroup.

The results of time dependent ROC analysis for BMI (reference), WC, VAI, and CVAI in relation to all participants, gender subgroups, and obesity subgroups is presented in Table 5. During the participant follow-ups (2, 4, 6, and 8 years), the CVAI showed higher levels in the AUC (95% CI) than BMI, whereas the VAI showed lower levels in the AUC (95% CI) than BMI. Similar findings were observed in the gender and obesity subgroups. In men, the AUC (95% CI) was higher in the CVAI than in BMI at the 4-year and 5-year follow-ups. In women, the AUC (95% CI) was consistently higher in the CVAI than in BMI across all follow-up times. In addition, women had a higher AUC (95% CI) for the CVAI than men across all follow-up times. In the obesity subgroups, the AUC (95% CI) for the CVAI was higher in the non-obese group than in the obese group across all follow-up times. The ROC curves determined by time dependent ROC analysis are shown in Supplementary Materials 1-5.

## DISCUSSION

The optimal index for assessing VAT remains debatable. It is known that anthropometric measurements are inherently limited in their ability to precisely measure VAT. Although the VAI has been used to predict VAT, its applicability in Asians is questionable. Thus, the CVAI was developed for the Chinese population to obtain a more precise assessment of VAT in Asians [12].

The present study was a large scale cohort study to verify the predictive ability of the CVAI for incident hypertension among working-aged Korean adults. In this study, the CVAI quartile levels were proportionally associated with the risk of hypertension. An identical association was observed in the 4 subgroups (men, women, obese, and non-obese). In particular, the CVAI was a more reliable predictor of hypertension than BMI, WC, and the VAI. These results indicated that increases in the CVAI strongly predicted the development of hypertension, even in relatively young Korean adults. This suggests that the CVAI is a reliable tool for estimating VAT to predict hypertension in working-aged Koreans.

Previous studies of middle-aged Chinese showed similar findings [17,18]. In a cross-sectional study of 34,732 Chinese [17], the CVAI was more significantly associated with hypertension (odds ratio, 3.475; 95% CI, 3.158 to 3.824) than other obesity indices such as the VAI, BMI, WC, and waist-to-height ratio. A stratified analysis showed that this association remained significant at any level of blood glucose, age, or estimated glomerular filtration rate. A prospective cohort study analyzed the relative risk (RR) of hypertension in relation to CVAI quartile levels in 10,304 Chinese with a median follow-up of 6.03 years [18]. In that study, the highest quartile group showed an increased RR for men (1.29; 95% CI, 1.05 to 1.59) and women (1.53; 95% CI, 1.22 to 1.91), when compared with the lowest quartile group. These studies support the potential of the CVAI as an early predictor of incident hypertension. However, although studies of middle-aged Chinese showed the predictive ability of the CVAI for the risk of hypertension, its generalizability and applicability to other Asians and younger age groups has not been well investigated. Our work expands the predictive ability of the CVAI for hypertension to other Asians and relatively young adults with our large sample size and comprehensive use of detailed variables. The CVAI may be a useful index to predict hypertension among young Asian adults and contribute to the prevention and early management of hypertension.

Our subgroup analyses further supported the predictive ability of the CVAI for cardiometabolic risk factors including hypertension. In the subgroup analysis by gender, the predictive ability of the CVAI was relatively stronger in women than men. Similar features of the CVAI have been observed in previous Chinese studies that reported higher levels of odds ratio and RR in women [17,18]. Gender differences in fat deposition may be a reason for this finding. It has been reported that obesity is more likely to contribute to the elevation of BP in women than men [23]. In particular, VAT was more strongly associated with hypertension in women than men [24]. Asian women are more predisposed to abdominal fat deposition [25], which can lead to a higher risk of adverse metabolic outcomes [26]. Thus, it seems that the high predictability of the CVAI derived from its precise assessment of VAT is particularly relevant in women. An elevated CVAI in women should be cautiously regarded as a warning sign of CV risk, requiring a preventive approach. Our obesity subgroup analysis addressed the ability of the CVAI to predict hypertension based on the degree of obesity. The non-obese group consistently showed higher HR levels for hypertension in each CVAI quartile group than the obese group. In addition, the AUC of the CVAI for hypertension was higher in the non-obese group than the obese group. These findings suggested that the CVAI was more effective at predicting hypertension in the non-obese group than the obese group. Results of the obesity subgroup analysis conflict with the common notion that obese people would have a higher CVAI predictability due to their greater VAT. The metabolic characteristics of Asians may explain these results. A characteristic of obesity in Asians is a relatively higher adiposity. Asians have 3% to 5% higher total body fat when compared with white Europeans with the same BMI [27]. Moreover, studies have demonstrated that Asians tend to accumulate more visceral fat [28]. It is presumed that this feature of Asians contributes to the higher cardiometabolic risk of Asians with the same metabolic conditions. Therefore, many Asians may have a relatively high degree of VAT despite a normal or low BMI and are at increased risk of hypertension. In practice, individuals with high body fat despite a normal body weight are considered to be ‘normal weight obese’, which is a typical phenotype of obesity in Asians [29]. Studies have demonstrated that individuals with normal weight obesity have an increased cardiometabolic risk and premature mortality [30,31]. Therefore, to predict and manage CV risk in Asians, it is clinically important to evaluate VAT in apparently non-obese individuals. As a reliable index for distinguishing normal weight obesity, the CVAI can be an effective tool for predicting CV risk in Asians.

Our analysis indicated that the CVAI was superior to BMI, WC, and the VAI in predicting hypertension. This finding has been consistently observed in other studies of Chinese adults. The plausible explanation for this finding may be the superiority of the CVAI in evaluating VAT among Asians. Studies have found that VAT is more strongly associated with incident hypertension than subcutaneous fat and other fat tissue [32,33]. Laboratory research has demonstrated that VAT is involved in the pathogenesis of hypertension through activation of sympathetic tone [34], elevated secretion of proinflammatory cytokines [33], disturbed vasodilation, and decreased natriuresis [35]. Therefore, it can be presumed that the predictability of indices for hypertension rely on the accuracy of VAT estimates. The CVAI more accurately estimates VAT than BMI, WC, and the VAI. BMI is unable to differentiate fat mass from lean mass and does not reflect fat distribution [36]. Although WC seems to be more precise in assessing central obesity than BMI, WC cannot distinguish subcutaneous fat from visceral fat. Iranian and Chinese studies found that the VAI was a weaker predictor of incident hypertension than other indices [37,38]. As an index reflecting both obesity and lipid levels, the CVAI was also significantly associated with VAT when assessed by computed tomography. When added to these reports, our results suggest the clinical usefulness of the CVAI in predicting cardiometabolic diseases related to VAT in Asians.

The present study had several limitations. First, the study sample only included Korean adults. Therefore, our results may not be generalizable to Asians living in other regions such as South Asia and Southeast Asia. Further studies should be conducted to include people living in other Asian areas. Second, the follow-up period (6.9 years) was not long enough to reflect the long-term incidence of hypertension. A longer follow-up period may result in a higher incidence of hypertension, which might lead to a difference in the magnitude of association between the obesity indices and the risk of hypertension. Third, we acknowledge that we were unable to consider all factors affecting the incidence of hypertension. The development of hypertension is the outcome of a complex interaction of biological, hereditary, genetic, and environmental factors, thus it was impossible to address all risk factors.

In conclusion, the CVAI was a reliable predictor of hypertension among working-aged Korean adults. In particular, the CVAI was superior to BMI, WC, and the VAI in predicting the development of hypertension. The predictive ability of the CVAI for hypertension was more prominent in women and non-obese individuals than in men and obese individuals, respectively. The CVAI can be used as a reliable and effective index for the early identification of Asians at high risk for hypertension.

## Figures and Tables

**Figure f1-epih-46-e2024034:**
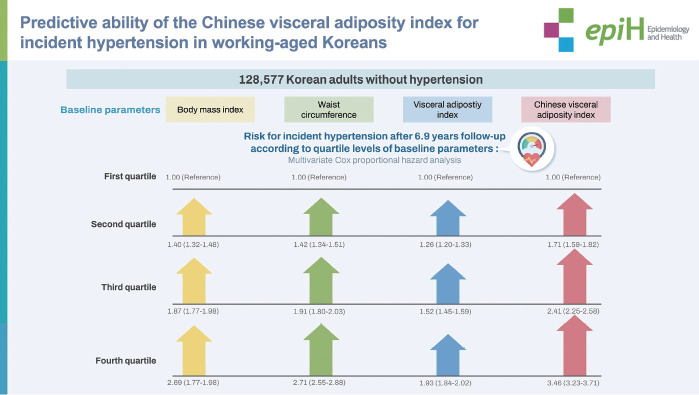


**Table 1.** The baseline clinical characteristics of subjects according to CVAI quartile (n=128,577)

Characteristics	Quartile 1	Quartile 2	Quartile 3	Quartile 4	p-value
Total (n)	32,145	32,144	32,144	32,144	
Men	4,765 (14.8)	15,002 (46.7)	25,527 (79.4)	29,994 (93.3)	<0.001
Age (yr)	35.1±4.7	38.4±6.0	39.6±6.8	40.5±7.0	<0.001
Systolic BP (mmHg)	100.6±10.1	106.2±10.8	111.4±10.2	115.3±9.8	<0.001
Diastolic BP (mmHg)	64.2±7.5	67.5±8.1	71.0±8.0	73.8±7.7	<0.001
Triglycerides (mmol/L)	0.7±0.3	1.0±0.4	1.4±0.6	1.9±1.1	<0.001
HDL-C (mmol/L)	1.8±0.4	1.6±0.3	1.4±0.3	1.2±0.3	<0.001
HOMA-IR	0.9±0.6	1.1± 0.6	1.3±1.0	1.9±1.2	<0.001
CRP	0.1±0.2	0.1±0.2	0.1±0.2	0.1±0.2	<0.001
BMI (kg/m^2^)	19.7±1.5	22.0±1.5	23.9±1.6	26.7±2.4	<0.001
Waist circumference (cm)	71.2±4.5	78.1±3.8	83.9±3.4	92.3±5.3	<0.001
VAI	0.7±0.3	1.0±0.6	1.4±1.0	2.4±1.8	<0.001
CVAI	7.8±13.2	42.4±9.0	73.2±8.9	113.9±20.6	<0.001
Range of CVAI	-59.8-26.7	26.7-57.8	57.8-89.0	89.0-260.0	
Average alcohol use (g/day)	7.3±13.9	11.0±17.7	16.5±22.2	21.2±25.8	<0.001
Current smoker	2,187 (6.8)	5,384 (16.7)	9,310 (29.0)	12,798 (39.8)	<0.001
High PA	4,808 (15.0)	5,940 (18.5)	5,724 (17.8)	5,042 (15.7)	<0.001
DM	129 (0.4)	302 (0.9)	801 (2.5)	1,827 (5.7)	<0.001
Incidence of hypertension	1,328 (4.1)	3,113 (9.7)	5,522 (17.2)	8,540 (26.6)	<0.001

Values are presented as mean±standard deviation or number (%). CVAI, Chinese visceral adiposity index; BP, blood pressure; HDL-C, high density lipoprotein-cholesterol; HOMA-IR, homeostatic model assessment for insulin resistance; CRP, c-reactive protein; BMI, body mass index; VAI, visceral adiposity index; PA, physical activity; DM, diabetes mellitus.

**Table 2.** Hazard ratio (HR) and 95% confidence intervals (CI) for hypertension according to BMI, waist circumference, VAI, and CVAI quartiles^1^

Characteristics	Quartile 1	Quartile 2	Quartile 3	Quartile 4
BMI (n)	33,317	32,505	30,905	31,850
Unadjusted HR	1.00 (reference)	1.97 (1.86, 2.09)	3.28 (3.11, 3.46)	5.22 (4.96, 5.49)
Model 1	1.00 (reference)	1.43 (1.35, 1.51)	1.95 (1.84, 2.06)	2.93 (2.77, 3.09)
Model 2	1.00 (reference)	1.40 (1.32, 1.48)	1.87 (1.77, 1.98)	2.69 (2.54, 2.84)
Incident cases, n (%)	1,800 (5.4)	3,414 (10.5)	5,215 (16.9)	8,074 (25.4)
Incidence density/PY	8.7/207,754	17.1/199,589	28.2/184,923	44.1/182,937
Range of BMI (kg/m^2^)	13.3-20.8	20.9-22.9	23.0-25.0	25.1-44.3
Waist circumference (n)	33,216	31,494	32,072	31,795
Unadjusted HR	1.00 (reference)	2.04 (1.93, 2.17)	3.49 (3.30, 3.68)	5.62 (5.33, 5.93)
Model 1	1.00 (reference)	1.45 (1.37, 1.55)	2.00 (1.88, 2.12)	2.97 (2.80, 3.15)
Model 2	1.00 (reference)	1.42 (1.34, 1.51)	1.91 (1.80, 2.03)	2.71 (2.55, 2.88)
Incident cases, n (%)	1,660 (5.0)	3,247 (10.3)	5,483 (17.1)	8,113 (25.5)
Incidence density/PY	8.1/205,869	16.7/194,051	28.4/193,140	44.5/182,143
Range of waist circumference (cm)	55.5-75.0	75.1-81.2	81.3-87.3	87.4-134.0
VAI (n)	32,145	32,144	32,144	32,144
Unadjusted HR	1.00 (reference)	1.37 (1.31, 1.44)	1.93 (1.84, 2.02)	2.95 (2.82, 3.09)
Model 1	1.00 (reference)	1.29 (1.22, 1.35)	1.58 (1.51, 1.66)	2.09 (2.00, 2.19)
Model 2	1.00 (reference)	1.26 (1.20, 1.33)	1.52 (1.45, 1.59)	1.93 (1.84, 2.02)
Incident cases, n (%)	2,642 (8.2)	3,614 (11.2)	4,998 (15.5)	7,249 (22.6)
Incidence density/PY	13.3/198,212	18.4/196,672	25.8/193,620	38.8/186,698
Range of VAI	0.119-0.681	0.681-1.028	1.028-1.659	1.659-47.620
CVAI (n)	32,145	32,144	32,144	32,144
Unadjusted HR	1.00 (reference)	2.36 (2.21, 2.51)	4.36 (4.11, 4.63)	7.34 (6.93, 7.78)
Model 1	1.00 (reference)	1.76 (1.65, 1.88)	2.57 (2.41, 2.76)	3.86 (3.61, 4.14)
Model 2	1.00 (reference)	1.71 (1.59, 1.82)	2.41 (2.25, 2.58)	3.46 (3.23, 3.71)
Incident cases, n (%)	1,328 (4.1)	3,113 (9.7)	5,522 (17.2)	8,540 (26.6)
Incidence density/PY	6.6/201,928	15.7/198,781	28.7/192,650	47.0/181,843
Range of CVAI	-59.8-26.7	26.7-57.8	57.8-89.0	89.0-260.0

BMI, body mass index; VAI, visceral adiposity index; CVAI, Chinese visceral adiposity index; PY, person-year; HOMA-IR, homeostatic model assessment for insulin resistance; CRP, c-reactive protein.

1 Model 1: age, gender, physical activity, alcohol intake, smoking, diabetes mellitus; Model 2: Model 1+HOMA-IR, CRP.

**Table 3.** Hazard ratio (HR) and 95% confidence intervals for hypertension according to BMI, waist circumference, VAI, and CVAI quartiles in men and women^1^

Characteristics	Quartile 1	Quartile 2	Quartile 3	Quartile 4
Men				
BMI (n)	19,063	18,831	19,179	18,215
Unadjusted HR	1.00 (reference)	1.38 (1.31, 1.45)	1.75 (1.67, 1.84)	2.54 (2.42, 2.66)
Model 1	1.00 (reference)	1.34 (1.28, 1.42)	1.67 (1.59, 1.76)	2.43 (2.32, 2.55)
Model 2	1.00 (reference)	1.30 (1.24, 1.37)	1.57 (1.49, 1.65)	2.15 (2.05, 2.26)
Incident cases, n (%)	2,515 (13.2)	3,334 (17.7)	4,162 (21.7)	5,416 (29.7)
Incidence density/PY	21.3/118,125	29.2/114,058	36.9/112,838	52.3/103,489
Range of BMI (kg/m^2^)	13.8-22.3	22.4-24.0	24.1-25.9	26.0-43.8
Waist circumference (n)	18,992	19,345	18,839	18,112
Unadjusted HR	1.00 (reference)	1.42 (1.35, 1.50)	1.75 (1.66, 1.84)	2.53 (2.41, 2.65)
Model 1	1.00 (reference)	1.37 (1.31, 1.45)	1.65 (1.57, 1.74)	2.38 (2.27, 2.49)
Model 2	1.00 (reference)	1.33 (1.26, 1.40)	1.55 (1.47, 1.63)	2.09 (1.99, 2.20)
Incident cases, n (%)	2,517 (13.3)	3,512 (18.2)	4,090 (21.7)	5,308 (29.3)
Incidence density/PY	21.3/118,027	30.0/116,962	36.8/111,219	51.9/102,301
Range of waist circumference (cm)	57.2-80.2	80.3-85.0	85.1-90.0	90.1-126.0
VAI (n)	18,822	18,822	18,822	18,822
Unadjusted HR	1.00 (reference)	1.34 (1.27, 1.40)	1.59 (1.52, 1.67)	2.11 (2.01, 2.21)
Model 1	1.00 (reference)	1.31 (1.24, 1.37)	1.53 (1.45, 1.60)	1.97 (1.87, 2.06)
Model 2	1.00 (reference)	1.26 (1.19, 1.32)	1.41 (1.34, 1.48)	1.72 (1.64, 1.80)
Incident cases, n (%)	2,679 (14.2%)	3,511 (18.7)	4,110 (21.8)	5,127 (27.2)
Incidence density/PY	23.2/115,696	30.9/113,545	36.8/111,661	47.6/107,608
Range of VAI	0.119-0.763	0.763-1.194	1.194-1.930	1.930-40.702
CVAI (n)	18,822	18,822	18,822	18,822
Unadjusted HR	1.00 (reference)	1.36 (1.29, 1.43)	1.83 (1.74, 1.92)	2.69 (2.57, 2.82)
Model 1	1.00 (reference)	1.31 (1.24, 1.38)	1.71 (1.63, 1.80)	2.45 (2.34, 2.58)
Model 2	1.00 (reference)	1.26 (1.20, 1.31)	1.60 (1.52, 1.68)	2.15 (2.04, 2.26)
Incident cases, n (%)	2,436 (12.9)	3,227 (17.1)	4,154 (22.1)	5,610 (29.8)
Incidence density/PY	20.7/117,790	28.0/115,061	37.5/110,890	53.5/104,768
Range of CVAI	-46.9-56.5	56.5-80.1	80.1-103.5	103.5-260.0
Women				
BMI (n)	13,849	13,433	13,204	12,803
Unadjusted HR	1.00 (reference)	1.40 (1.23, 1.59)	1.98 (1.75, 2.24)	4.14 (3.70, 4.63)
Model 1	1.00 (reference)	1.27 (1.11, 1.45)	1.68 (1.48, 1.90)	3.22 (2.88, 3.61)
Model 2	1.00 (reference)	1.26 (1.10, 1.43)	1.64 (1.45, 1.86)	3.04 (2.71, 3.41)
Incident cases, n (%)	395 (2.9)	537 (4.0)	735 (5.6)	1,409 (11.0)
Incidence density/PY	4.6/86,478	6.4/83,451	9.1/80,850	18.6/75,913
Range of BMI (kg/m^2^)	13.3-19.5	19.6-21.0	21.1-22.9	23.0-43.3
Waist circumference (n)	13,411	13,616	13,012	13,250
Unadjusted HR	1.00 (reference)	1.29 (1.13, 1.46)	1.67 (1.48, 1.89)	3.43 (3.07, 3.83)
Model 1	1.00 (reference)	1.19 (1.05, 1.36)	1.45 (1.28, 1.64)	2.72 (2.43, 3.04)
Model 2	1.00 (reference)	1.18 (1.04, 1.34)	1.42 (1.26, 1.61)	2.56 (2.29, 2.86)
Incidence cases, n (%)	412 (3.1)	565 (4.2)	699 (5.4)	1,400 (10.6)
Incidence density/PY	5.0/82,555	6.7/84,692	8.7/80,106	17.6/79,340
Range of waist circumference (cm)	55.5-70.5	70.6-75.0	75.1-80.1	80.2-134.0
VAI (n)	13,323	13,322	13,322	13,322
Unadjusted HR	1.00 (reference)	1.23 (1.09, 1.39)	1.52 (1.35, 1.71)	2.82 (2.54, 3.14)
Model 1	1.00 (reference)	1.19 (1.05, 1.34)	1.39 (1.24, 1.56)	2.36 (2.12, 2.63)
Model 2	1.00 (reference)	1.17 (1.04, 1.32)	1.35 (1.20, 1.52)	2.21 (1.98, 2.46)
Incidence cases, n (%)	469 (3.5%)	585 (4.4)	726 (5.5)	1,296 (9.7)
Incidence density/PY	5.7/82,077	7.1/82,349	8.8/82,292	16.2/79,975
Range of VAI	0.139-0.613	0.613-0.860	0.860-1.288	1.288-47.623
CVAI (n)	13,323	13,322	13,322	13,322
Unadjusted HR	1.00 (reference)	1.45 (1.26, 1.67)	2.10 (1.84, 2.39)	5.36 (4.76, 6.05)
Model 1	1.00 (reference)	1.29 (1.12, 1.49)	1.72 (1.50, 1.97)	3.84 (3.36, 4.37)
Model 2	1.00 (reference)	1.27 (1.10, 1.47)	1.67 (1.45, 1.91)	3.55 (3.11, 4.06)
Incidence cases, n (%)	322 (2.4)	486 (3.7)	688 (5.2)	1,580 (11.9)
Incidence density/PY	3.9/83,201	5.8/84,236	8.4/82,374	20.6/76,882
Range of CVAI	-59.8-8.5	8.5-25.7	25.7-46.9	46.9-220.0

BMI, body mass index; VAI, visceral adiposity index; CVAI, Chinese visceral adiposity index; PY, person-year; HOMA-IR, homeostatic model assessment for insulin resistance; CRP, c-reactive protein.

1 Model 1: age, physical activity, alcohol intake, smoking, total calorie intake; Model 2: Model 1+HOMA-IR, CRP.

**Table 4.** Hazard ratio (HR) and 95% confidence intervals for hypertension according to BMI, waist circumference, VAI, and CVAI quartiles in obese and non-obese subgroups^1^

Characteristics	Quartile 1	Quartile 2	Quartile 3	Quartile 4
Obese group (BMI ≥25 kg/m^2^)				
Waist circumference (n)	8,403	8,488	8,372	7,878
Unadjusted HR	1.00 (reference)	1.20 (1.13, 1.29)	1.45 (1.36, 1.54)	1.85 (1.74, 1.97)
Model 1	1.00 (reference)	1.11 (1.04, 1.18)	1.31 (1.22, 1.39)	1.70 (1.60, 1.81)
Model 2	1.00 (reference)	1.09 (1.02, 1.17)	1.27 (1.19, 1.36)	1.62 (1.52, 1.72)
Incident cases, n (%)	1,627 (19.4)	1,949 (23.0)	2,236 (26.7)	2,518 (32.0)
Incidence density/PY	32.7/49,750	39.4/49,525	46.5/48,051	58.2/43,228
Range of waist circumference (cm)	64.3-87.8	87.9-91.0	91.1-95.0	95.1-134.0
VAI (n)	8,286	8,285	8,285	8,285
Unadjusted HR	1.00 (reference)	1.20 (1.13, 1.29)	1.35 (1.26, 1.43)	1.63 (1.53, 1.74)
Model 1	1.00 (reference)	1.18 (1.10, 1.26)	1.29 (1.21, 1.38)	1.54 (1.44, 1.64)
Model 2	1.00 (reference)	1.16 (1.08, 1.24)	1.25 (1.17, 1.34)	1.45 (1.36, 1.55)
Incident cases, n (%)	1,663 (20.1)	1,971 (23.8)	2,153 (26.0)	2,543 (30.7)
Incidence density/PY	34.1/48,698	41.0/48,055	45.4/47,413	54.8/46,388
Range of VAI	0.153-1.063	1.063-1.617	1.617-2.503	2.503-47.623
CVAI (n)	8,286	8,285	8,285	8,285
Unadjusted HR	1.00 (reference)	1.41 (1.31, 1.51)	1.79 (1.68, 1.92)	2.37 (2.22, 2.53)
Model 1	1.00 (reference)	1.22 (1.13, 1.31)	1.49 (1.39, 1.60)	1.92 (1.79, 2.07)
Model 2	1.00 (reference)	1.19 (1.11, 1.28)	1.44 (1.34, 1.55)	1.80 (1.68, 1.94)
Incident cases, n (%)	1,370 (16.5)	1,890 (22.8)	2,280 (27.5)	2,790 (33.7)
Incidence density/PY	27.5/49,909	38.7/48,808	48.4/47,127	62.4/44,710
Range of CVAI	-1.6-88.4	88.4-105.5	105.5-123.5	123.5-260.5
Non-obese group (BMI <25 kg/m^2^)				
Waist circumference (n)	24,495	23,330	23,884	23,727
Unadjusted HR	1.00 (reference)	1.70 (1.58, 1.83)	2.79 (2.61, 2.99)	4.11 (3.84, 4.39)
Model 1	1.00 (reference)	1.25 (1.16, 1.36)	1.60 (1.49, 1.72)	1.95 (1.81, 2.10)
Model 2	1.00 (reference)	1.23 (1.13, 1.32)	1.53 (1.42, 1.65)	1.80 (1.67, 1.94)
Incident cases, n (%)	1,091 (4.5)	1,823 (7.8)	3,032 (12.7)	4,227 (17.8)
Incidence density/PY	7.2/151,619	12.6/144,760	20.7/146,699	29.9/141,571
Range of waist circumference (cm)	55.5-73.0	73.1-78.1	78.2-82.8	82.9-110.0
VAI (n)	23,859	23,859	23,859	23,859
Unadjusted HR	1.00 (reference)	1.22 (1.15, 1.30)	1.52 (1.43, 1.62)	2.25 (2.12, 2.38)
Model 1	1.00 (reference)	1.21 (1.14, 1.29)	1.37 (1.29, 1.45)	1.70 (1.61, 1.81)
Model 2	1.00 (reference)	1.18 (1.11, 1.26)	1.31 (1.23, 1.39)	1.57 (1.48, 1.66)
Incident cases, n (%)	1,707 (7.2)	2,104 (8.8)	2,621 (11.0)	3,741 (15.7)
Incidence density/PY	11.6/147,540	14.3/147,405	17.9/146,612	26.1/143,091
Range of VAI	0.119-0.621	0.621-0.894	0.894-1.362	1.362-33.700
CVAI (n)	23,859	23,859	23,859	23,859
Unadjusted HR	1.00 (reference)	1.83 (1.68, 1.99)	3.53 (3.27, 3.80)	5.69 (5.29, 6.12)
Model 1	1.00 (reference)	1.41 (1.30, 1.54)	2.01 (1.85, 2.18)	2.53 (2.32, 2.76)
Model 2	1.00 (reference)	1.37 (1.26, 1.49)	1.90 (1.75, 2.07)	2.30 (2.11, 2.51)
Incident cases, n (%)	873 (3.7)	1,638 (6.9)	3,046 (12.8)	4,616 (19.3)
Incidence density/PY	5.8/149,555	10.9/149,839	20.9/145,460	33.0/139,793
Range of CVAI	-59.8-18.4	18.4-42.1	42.1-66.8	66.8-152.8

BMI, body mass index; VAI, visceral adiposity index; CVAI, Chinese visceral adiposity index; PY, person-year; HOMA-IR, homeostatic model assessment for insulin resistance; CRP, c-reactive protein.

1 Model 1: age, gender, physical activity, alcohol intake, smoking, diabetes mellitus; Model 2: Model 1+HOMA-IR, CRP.

**Table 5.** Time dependent area under the ROC curve with 95% confidence interval for BMI, WC, VAI, and CVAI at the 2-, 4-, 6-, and 8-year follow-ups for subjects overall and 4 subgroups

Variables	BMI	WC	VAI	CVAI
All participants (yr)				
2	0.695 (0.668, 0.722)	0.700 (0.673, 0.727)	0.614 (0.584, 0.646)^***^	0.718 (0.693, 0.744)^**^
4	0.698 (0.680, 0.716)	0.697 (0.680, 0.715)	0.640 (0.620, 0.660)^***^	0.725 (0.708, 0.742)^***^
6	0.699 (0.683, 0.714)	0.700 (0.685, 0.716)	0.643 (0.626, 0.660)^***^	0.725 (0.710, 0.739)^***^
8	0.659 (0.639, 0.678)	0.655 (0.635, 0.674)	0.587 (0.567, 0.608)^***^	0.672 (0.653, 0.691)^**^
Men (yr)				
2	0.630 (0.594, 0.666)	0.629 (0.594, 0.665)	0.604 (0.570, 0.638)	0.642 (0.607, 0.676)
4	0.641 (0.619, 0.664)	0.644 (0.622, 0.666)	0.625 (0.602, 0.647)	0.663 (0.642, 0.684)^***^
6	0.644 (0.624, 0.663)	0.643 (0.623, 0.662)	0.622 (0.602, 0.642)	0.662 (0.643, 0.681)^**^
8	0.616 (0.592, 0.644)	0.614 (0.589, 0.638)	0.602 (0.578, 0.626)	0.623 (0.599, 0.647)
Women (yr)				
2	0.628 (0.523, 0.733)	0.606 (0.512, 0.700)	0.623 (0.517, 0.730)	0.703 (0.617, 0.790)^*^
4	0.662 (0.606, 0.717)	0.642 (0.587, 0.697)	0.665 (0.604, 0.726)	0.727 (0.673, 0.781)^***^
6	0.672 (0.628, 0.715)	0.660 (0.617, 0.703)	0.671 (0.627, 0.715)	0.724 (0.682, 0.766)^***^
8	0.630 (0.584, 0.676)	0.614 (0.567, 0.660)	0.584 (0.539, 0.629)	0.655 (0.612, 0.699)^*^
Obese (yr)				
2	0.583 (0.536, 0.629)	0.605 (0.561, 0.649)	0.535 (0.492, 0.579)	0.631 (0.589, 0.674)^*^
4	0.569 (0.539, 0.599)	0.591 (0.562, 0.621)	0.556 (0.526, 0.585)	0.622 (0.594, 0.650)^***^
6	0.575 (0.548, 0.601)	0.594 (0.568, 0.620)	0.548 (0.522, 0.574)	0.624 (0.599, 0.649)^***^
8	0.586 (0.551, 0.621)	0.592 (0.557, 0.628)	0.569 (0.534, 0.605)	0.619 (0.584, 0.654)^*^
Non-obese (yr)				
2	0.665 (0.623, 0.706)	0.700 (0.661, 0.740)^*^	0.642 (0.595, 0.688)	0.742 (0.704, 0.779)^***^
4	0.682 (0.656, 0.708)	0.679 (0.653, 0.704)	0.618 (0.589, 0.647)^***^	0.716 (0.691, 0.741)^***^
6	0.672 (0.650, 0.694)	0.666 (0.644, 0.688)	0.618 (0.594, 0.642)^***^	0.701 (0.680, 0.723)^***^
8	0.620 (0.594, 0.645)	0.625 (0.600, 0.650)	0.560 (0.534, 0.587)^***^	0.657 (0.632, 0.681)^***^

Reference: BMI. ROC, receiver operator characteristic; BMI, body mass index; WC, waist circumference; VAI, visceral adiposity index; CVAI, Chinese visceral adiposity index.

* p<0.05,

** p<0.01,

*** p<0.001.

## Supplementary materials

Supplementary Material 1. ROC curve in all participants

Supplementary Material 2. ROC curve in men

Supplementary Material 3. ROC curve in women

Supplementary Material 4. ROC curve in obese subgroup

Supplementary Material 5. ROC curve in non-obese subgroup
