# Supplementary material for: Predictive ability of the Chinese visceral adiposity index for incident hypertension in working-aged Koreans
Source: Epidemiol Health. 2024 Feb 27;46:e2024034. doi: 10.4178/epih.e2024034 (PMC11176715; doi:10.4178/epih.e2024034)
Supplement: Supplementary Material 5. — ROC curve in non-obese subgroup [file epih-46-e2024034-Supplementary-5.pptx]

## Slide 1
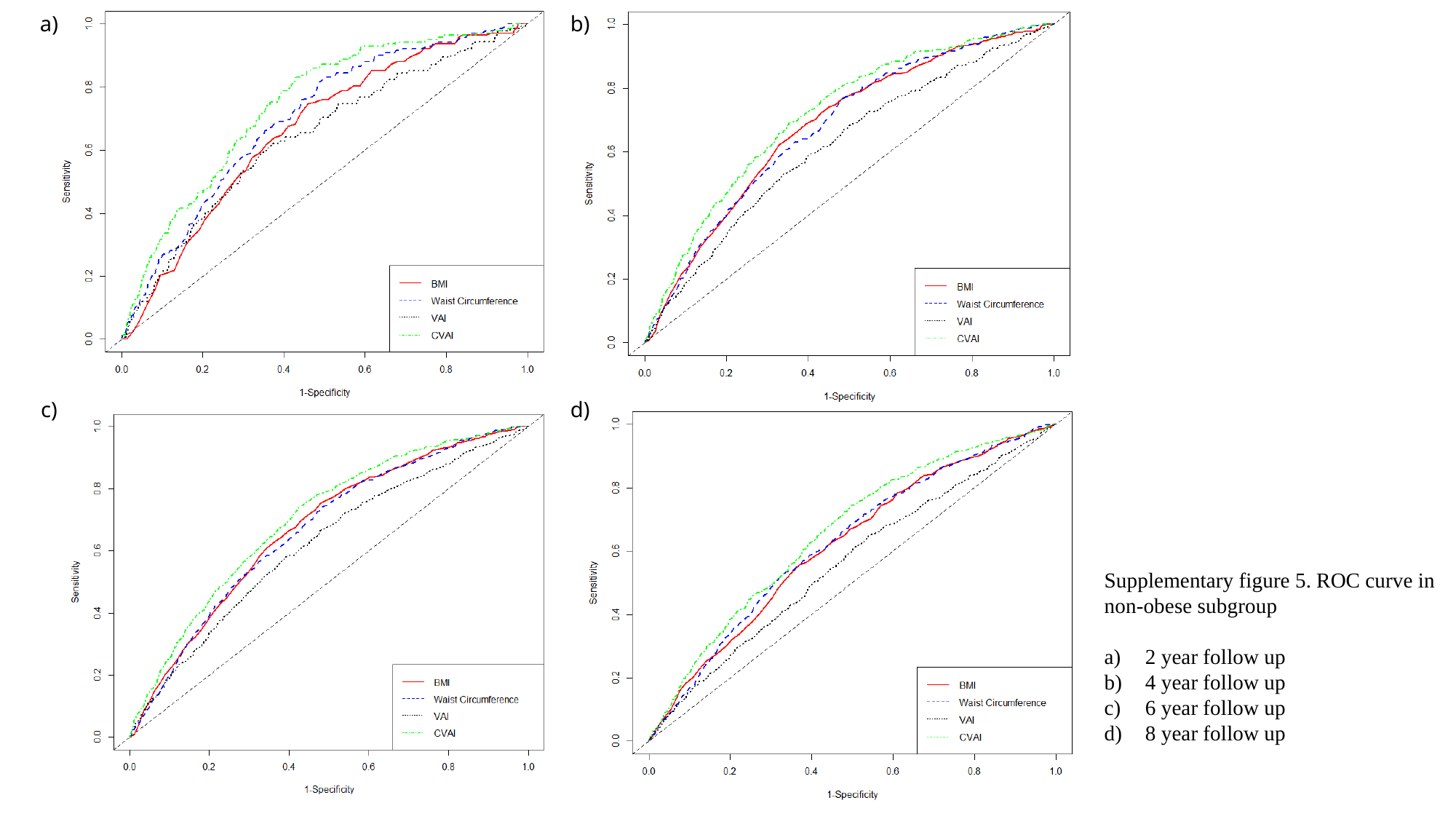

a)
b)
c)
d)
Supplementary figure 5. ROC curve in non-obese subgroup
2 year follow up
4 year follow up
6 year follow up
8 year follow up
